# Introducing Simple Detection of Bioavailable Arsenic at Rafaela (Santa Fe Province, Argentina) Using the ARSOlux Biosensor

**DOI:** 10.3390/ijerph120505465

**Published:** 2015-05-21

**Authors:** Konrad Siegfried, Sonja Hahn-Tomer, Andreas Koelsch, Eva Osterwalder, Juergen Mattusch, Hans-Joachim Staerk, Jorge M. Meichtry, Graciela E. De Seta, Fernando D. Reina, Cecilia Panigatti, Marta I. Litter, Hauke Harms

**Affiliations:** 1 Helmholtz Centre for Environmental Research GmbH—UFZ, Department Environmental Microbiology, Permoserstrasse 15, Leipzig 04318, Germany; E-Mails: sonja.hahn-tomer@ufz.de (S.H.T.); andreas.koelsch@ufz.de (A.K.); eva.osterwalder@ufz.de (E.O.); hauke.harms@ufz.de (H.H.); 2 Helmholtz Centre for Environmental Research GmbH—UFZ, Department Analytical Chemistry, Permoserstrasse 15, Leipzig 04318, Germany; E-Mails: juergen.mattusch@ufz.de (J.M.); ha-jo.staerk@ufz.de (H.-J.S.); 3 Facultad Regional Buenos Aires, Universidad Tecnologica Nacional, Medrano 951, Argentina; E-Mails: martinjmyque@gmail.com (J.M.M.); egdeseta@yahoo.com.ar (G.E.D.S.); fernando-reina@hotmail.com (F.D.R.); 4 Consejo Nacional de Investigaciones Científicas y Técnicas, Rivadavia 1917, Argentina; E-Mail: marta.litter@gmail.com; 5 Facultad Regional Rafaela, Universidad Tecnologica Nacional, M. Acuña 49, Rafaela 2300, Argentina; E-Mail: cecipanigatti@hotmail.com; 6 Gerencia Química, Centro Atómico Constituyentes, Comisión Nacional de Energía Atómica, Av. Gral. Paz 1499, San Martín 1650, Argentina; 7 Instituto de Investigación e Ingeniería Ambiental, Universidad de General San Martín, Peatonal Belgrano 3563, San Martín 1650, Argentina

**Keywords:** bioavailable arsenic, biosensor, ground water, Argentina, Chaco Pampean plain

## Abstract

Numerous articles have reported the occurrence of arsenic in drinking water in Argentina, and the resulting health effects in severely affected regions of the country. Arsenic in drinking water in Argentina is largely naturally occurring due to elevated background content of the metalloid in volcanic sediments, although, in some regions, mining can contribute. While the origin of arsenic release has been discussed extensively, the problem of drinking water contamination has not yet been solved. One key step in progress towards mitigation of problems related with the consumption of As-containing water is the availability of simple detection tools. A chemical test kit and the ARSOlux biosensor were evaluated as simple analytical tools for field measurements of arsenic in the groundwater of Rafaela (Santa Fe, Argentina), and the results were compared with ICP-MS and HPLC-ICP-MS measurements. A survey of the groundwater chemistry was performed to evaluate possible interferences with the field tests. The results showed that the ARSOlux biosensor performed better than the chemical field test, that the predominant species of arsenic in the study area was arsenate and that arsenic concentration in the studied samples had a positive correlation with fluoride and vanadium, and a negative one with calcium and iron.

## 1. Introduction

### 1.1. The Problem of Arsenic in the World and in Argentina 

Occurrence of arsenic in drinking water has been reported as being a problem in different parts of the world. This is especially true for populations in isolated and less developed regions where contaminated water is used widely as a drinking water supply and is the main source of arsenic exposure [1,2,3]. While more than 100 million people in China, South- and Southeast Asia are affected [4], in Latin America it is estimated that 14 million people live in regions with elevated arsenic water contamination [5,6]. In Argentina several settlements in the Chaco-Pampean Plain are not connected to any raw water treatment system. In some cases, the available technologies are not suited to treat water contaminated with arsenic and other associated toxic compounds, such as boron and fluorine [2,5,6,7,8,9].

### 1.2. Commonly Used Detection Methods and Their Limitations

A sustainable introduction of treatment technologies should be based on a countrywide system for the monitoring of the water quality of treatment systems as well as public and private wells. Arsenic in water samples is commonly evaluated by ICP-MS, AAS and chemical test kits based on the Gutzeit method [10,11]. While ICP-MS, ICP-OES, AAS and other spectrometric analyses lead to highly accurate results, they require large investments, installation infrastructure (gas, chemical supplies) and highly skilled experts to correctly execute the tests and interpret the results. In many less developed regions throughout the world, the described detection methods are not available at all. On the other hand, the chemical field test kits are low-cost and simple to handle, but they include hazardous chemicals (HgBr_2_) and create highly toxic arsenic compounds (AsH_3_) and wastes that have to be disposed of properly. For some of these kits it is questioned if they can yield results with the required accuracy and precision [11,12]. Nowadays some new, simple and environmentally friendly methods, such as stripping voltammetry and biosensors, offer sustainable measurement procedure alternatives.

### 1.3. Arsenic Biosensor Measurement

The ARSOlux biosensor test kit, developed by scientists at the Helmholtz Centre for Environmental Research—UFZ (Germany) and the Université de Lausanne (Switzerland), is based on the non-pathogenic genetically modified bioreporter bacteria strain *E.coli* DH5α that can detect bioavailable total arsenic concentrations in ground and surface water samples [13,14,15]. The whole cell bioreporter bacteria respond to arsenic by synthesizing bacterial luciferase resulting in a blue light emission [13]. The bioluminescence signal measured by a luminometer correlates linearly with arsenic concentration up to a level of 80 µg/L. At higher concentrations ranging from 80–200 µg/L a non-linear functional relation is used to calculate the arsenic concentration from light emission data (relative light units) by extra- and interpolation. The limit of detection (LOD) lies at 5 µg/L. Therefore the ARSOlux biosensor field kit is able to accurately detect arsenic concentrations at the World Health Organisation (WHO) recommended acceptable limit for drinking water of 10 µg/L arsenic [16]. A special feature of the method is the ability to measure many samples in parallel without using toxic chemicals. The biological test kit can measure bioavailable total dissolved arsenic. Arsenic adsorbed on ferric oxyhydroxides and other undissolved arsenic compounds are not bioavailable and cannot be detected by the bioreporter bacteria [13]. The luminometer device measures and stores quantitative values that are easily transferred to a laptop using custom software.

## 2. Materials and Methods

### 2.1. Site Description and Water Sampling

**Figure 1:**
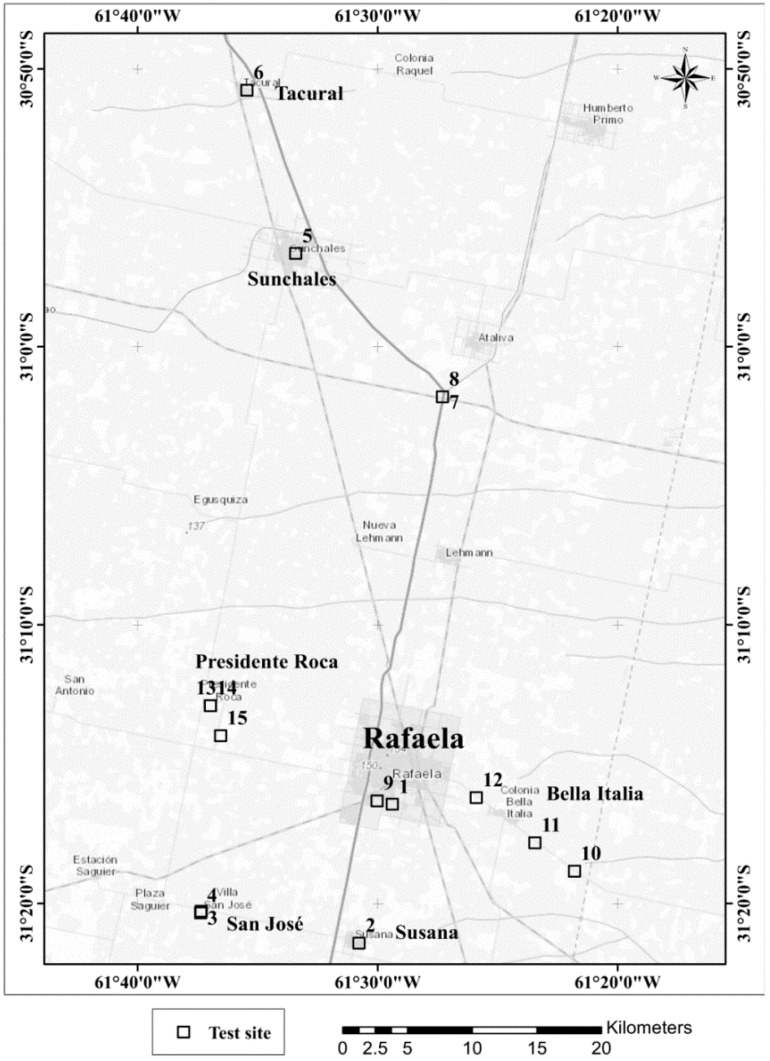
Sampling sites near Rafaela (Santa Fe Province, Argentina).

The study was conducted in the Argentine part of the Chaco-Pampean Plain near the city of Rafaela, Santa Fe province (Figure 1). Several authors have reported moderate to very high arsenic concentrations in the ground water of this particular area; however, the data on As levels in this region are rather scarce when compared to other places in the Chaco-Pampean plain [5,17,18].

During the campaign water samples were taken from wells of 15 different locations in Rafaela (92 m above sea level (asl), 31.273594° S; 61.489766° W) and surrounding towns in Susana (90 m asl, 31.357068° S; 61.513025° W), San José (102 m asl, 31.338839° S; 61.622951 W), Sunchales (97 m asl, 30.943634° S; 61.556958° W), Tacural (98 m asl, 30.845649° S; 61.590777° W), Bella Italia (75 m asl, 31.314193° S; 61.363348° W), Presidente Roca (103 m asl, 31.215263 S; 61.616112° W) and a dairy farm well (97 m asl, 31.02950° S, 61.45498° W) in Santa Fe Province. Coordinates of the sampled wells were recorded with a Garmin eTrex 10 GPS device (Garmin, Southampton, U.K.; Appendix, Table A1). The ground water to be tested was first collected from the wells in a 10 L bucket; temperature, pH, total dissolved solids (TDS), electrical conductivity (EC), salinity and dissolved oxygen (DO) were determined on site by a multi-parameter probe (WTW, Multi 3430 SET G, Weilheim, Germany; Appendix, Table A2). A 50 mL sample was filtered with a 0.80 µm cellulose acetate filter and stabilized with HNO_3_ to a final concentration of 10 mM for subsequent laboratory analysis of total concentrations of arsenic and other elements with ICP-MS, ICP-OES at the Department of Analytical Chemistry of the UFZ in Germany [19]. A 1 mL sample was filtered, acidified with H_3_PO_4_ to a final concentration of 10 mM and filled into darkened vials for analysis of arsenate and arsenite with HPLC-ICP-MS at the UFZ in Germany [20]. Samples analysed with the ARSOlux biosensor were filtered, transported to the laboratory of the Regional Faculty of the National Technological University (UTN) in Rafaela and analysed on the same day of sampling. The same samples were also analysed for total arsenic with a chemical field test kit (Econo Quick^™^, ITS, Rock HilL, SC, USA). The analyses with the chemical test kit were performed in duplicate immediately after on-site sampling.

### 2.2. Water Testing with the ARSOlux Biosensor

In order to test a sample with the ARSOlux biosensor for the total arsenic concentration, a volume of 1 mL is taken from the water source or sample flask with a syringe. If the water shows high turbidity and red colour (elevated iron concentration), it is recommended to filter the sample with a 0.45–0.80 µm cellulose acetate filter. The septum rubber stopper of the biosensor vial is penetrated with the needle of the syringe and the sample is emptied into the vial. The lyophilisate containing the freeze-dried bioreporter bacteria is re-suspended and the bacteria are revitalised. The biosensor vial is marked with the sample number on the septum stopper or crimp seal. When a certain number of biosensors are filled with water samples, the time must be recorded. To avoid time delays a maximum of 20–30 sensors should be incubated at once. After the incubation start of the first lot, the next lot can be incubated after 15 min and so on. The filled biosensors must be incubated for exactly 2 h at a temperature of 30 °C to restart the metabolism of the bacteria. After the incubation period of 2 h, the biosensor vial is inserted into the measuring channel of a luminometer device to measure the light emission of the bacteria. The measurement itself takes only 10 s and the arsenic concentration is displayed on the screen of the measuring device. The luminometer device must be calibrated prior to the measurement of samples. This is done with biosensors previously incubated with arsenic standards of 5, 20, 50 and 200 µg/L.

### 2.3. Specific Considerations/Preferences for the Correct Use of the Biosensor

**Figure 2:**
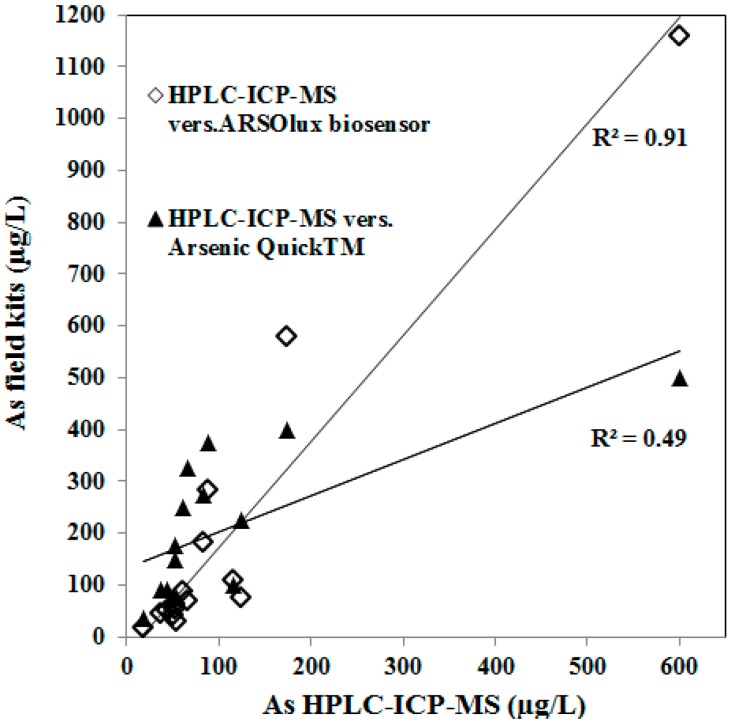
Cross comparison of total arsenic concentration detected by HPLC-ICP-MS and the field kits ARSOlux and Arsenic Quick^™^.

The measuring range of the ARSOlux biosensor is from 5 to 200 μg/L arsenic. The optimum incubation conditions comprise a temperature of 30 °C (between 20 °C (68 °F) and 37 °C (99 °F)), a pH of 6–8, salinity ≤ 0.5% and an electric conductivity ≤ 9 mS/cm (TDS ≤ 9 g/L). For best results a mobile or stationary incubator is recommended, which guarantees a constant temperature of 30 °C. Arsenic standards used for calibration of the luminometer device should be prepared shortly before the execution of the actual measurements. Unfiltered samples must be analysed immediately after sampling to prevent adverse effects of adsorption and co-precipitation of arsenic. Samples should not be acidified or chlorinated before the measurement. In the case of very high arsenic concentrations, two biosensor vials are required to analyse one sample since high arsenic concentration of >200 μg/L can inhibit the light response of the bacteria and hence lead to false negative results. In this case, the first vial is filled with the undiluted sample and the second vial with a diluted sample (dilution factor 10). If the result of the diluted sample is higher than the result of the undiluted sample, than the result of the diluted sample has to be multiplied by the dilution factor (10). In such a case the result of the undiluted sample is disregarded. By using the described procedure, concentrations up to 2000 µg/L As can be detected with satisfying accuracy (Figure 2). After completing all measurements the used genetically modified bioreporter bacteria inside the biosensor vials must be deactivated by using a disinfectant. Accordingly, the syringe is filled with the disinfectant, the septum stopper is penetrated and one drop of the disinfectant is added to each of the used biosensor vials. The used and deactivated biosensors, syringes, needles and other waste are collected in plastic bags and a container, which finally is autoclaved.

### 2.4. Water Testing with the Arsenic Quick^™^ Kit

The chemical Arsenic Quick^™^ field test kit (ITS, Rock Hill, SC, USA), based on the Gutzeit method, can semi-quantitatively detect total arsenic concentration in aqueous samples [21]. A 100 mL sample must be filled into a plastic reaction bottle. Thereafter, three spoons of the first reagent containing a mixture of tartaric acid (98.7%), ferrous sulphate (0.7%) and nickel sulphate (0.6%) must be added to the sample, which has to be agitated for 15 s. After that, two spoons of the second reagent potassium peroxymonopersulfate is added, agitated and the sample mixture is left standing for 2 min.

Finally, three spoons of the third reagent zinc are added and the mixture agitated for 5 s. A mercury bromide test stripe is inserted into a turret and the reaction bottle is capped with the turret and left for 10 min. After 10 min the test stripe is removed from the turret and the colour on the stripe is compared to a colour scale. The result of the test can give a semi-quantitative result of the arsenic concentration range (<5, ≥5, ≥10, ≥20, ≥30, ≥40, ≥50, ≥60, ≥80, ≥100, ≥150, ≥200, ≥250, ≥300, ≥400, >500 µg/L As). To compare the semi-quantitative results of the Arsenic Quick^™^ kit with the other used arsenic detection methods in this study, the results of two replicates were averaged (Appendix, Table A3).

### 2.5. Chemical Analysis

Total concentrations of arsenic (As), aluminium (Al), boron (B), calcium (Ca), chromium (Cr), iron (Fe), manganese (Mn), silicon (Si) and vanadium (V) were determined with inductively coupled plasma optical emission spectrometry (ICPOES, CIROS, Spectro A.I., Kleve, Germany), in some instances operated as flow injection analysis (FIA), and inductively coupled plasma quadrupole mass spectrometry (ICP-MS, ELAN 6000 DRC-e, Perkin-Elmer, Waltham, MA, USA) were applied for arsenic concentrations below and above 100 μg/L, respectively (Appendix, Table A4). The differentiation of arsenic species was performed by coupling HPLC online with ICP-MS at the laboratory of the Department of Analytical Chemistry of the UFZ in Leipzig, Germany [16] (Appendix, Table A3). Total inorganic carbon (TIC) and total organic carbon (TOC) were analysed with a 5000-A TOC analyser (Shimadzu, Kyoto, Japan) in the laboratory of the Gerencia Química, CNEA, Buenos Aires, Argentina. Fluoride (F^−^), nitrate (NO_3_^−^), nitrite (NO_2_^−^), ammonium (NH_4_^+^), chloride (Cl^−^), bicarbonate (HCO_3_^−^) and sulphate (SO_4_^2−^) were measured at the National Technological University (UTN), Regional Faculty Rafaela, Santa Fe, Argentina (Appendix, Table A5). F^−^ and NO_3_^−^ were analysed with an ion-selective electrode according to [22,23], respectively. NO_2_^−^ and NH_4_^+^ were analysed with a colorimetric method according to [24,25], respectively. Cl^−^ was analysed by titration with silver nitrate [26], HCO_3_^−^ was analysed by titration [27] and SO_4_^2−^ was measured by turbidimetry [28].

### 2.6. Correlation Analysis

The calculation of the Pearson and the Spearman correlation coefficients between total arsenic concentration and the other analysed parameters (pH, conductivity, element and ion concentrations) was done using the Origin 8.0 software (OriginLab Corporation, Northampton, MA, USA).

## 3. Results

### 3.1. Correlations

The arsenic concentrations of all tested wells ranged from 16 to 2000 µg/L (ICP-MS) and were thus above the World Health Organization’s threshold for drinking water [29] (guideline value: 10 µg/L As); also, many of them were above the 50 µg/L established by the local legislation of Santa Fe province [30]. Of the other analysed parameters, boron and fluoride were detected at concentrations above the WHO guideline values of 2.4 mg/L and 1.5 mg/L [16], respectively. Although no limit is set by the WHO or the Argentine legislation for V in drinking water, the EPA has set vanadium as one of 30 potential unregulated contaminants for further evaluation [31], as it has been identified as a possible carcinogen [32]; the California Department of Public Health has established a notification level of 50 μg/L for V in drinking water [33], a value that was largely exceeded by samples of many wells here studied. The measured oxidation/reduction potential (ORP) values are somewhat lower than those previously reported for groundwater of the Chaco-Pampean plain (100 < ORP < 525 mV [17]); the other parameters are in good agreement with recently reported values for the Rafaela region [18].

**Table 1:** Pearson (2-tailed) and spearman correlation coefficients for total arsenic and analysed water quality parameters.

Parameter	Pearson Correlation Coefficient	Significance	Spearman Correlation Coefficient	Significance
pH	0.601	0.018	0.835	0.00011
EC	−0.107	0.705	−0.557	0.031
DO	−0.443	0.098	−0.361	0.187
IC	0.344	0.209	0.246	0.376
TOC	−0.439	0.102	−0.486	0.0664
Al	−0.147	0.602	−0.258	0.354
B	0.570	0.026	0.259	0.351
Ca	−0.288	0.298	−0.836	0.0001
Cr	0.058	0.838	−0.091	0.746
Fe	−0.307	0.265	−0.721	0.0024
Mn	−0.201	0.472	−0.611	0.0156
Si	−0.559	0.0302	−0.753	0.0012
V	0.960	<0.0001	0.909	<0.0001
F^−^	0.687	0.0066	0.663	0.0011
NO_3_^−^	−0.214	0.462	−0.499	0.0694
Cl^−^	−0.132	0.652	−0.574	0.032
HCO_3_^−^	0.331	0.228	0.229	0.412
SO_4_^2−^	−0.104	0.712	−0.55	0.0337

All of the samples showed elevated results for EC, TDS and salinity. A positive correlation of arsenic was observed for results derived from ICP-MS with concentrations of boron (r = 0.57, *n* = 15), vanadium (r = 0.96) and pH (r = 0.60). A negative correlation of arsenic (ICP-MS) and silicate was observed (r = −0.56, *n* = 15). Arsenite (AsIII) concentrations measured by HPLC-ICP-MS correlated with the total concentration of iron (r = 0.56, *n* = 15) and manganese (r = 0.55, *n* = 15).

From Table 1, it can be observed that, using the Pearson correlation [8], a significant correlation with As concentration at a 95% of confidence (*p* < 0.05) can be observed for pH and the concentration of B, Si, V and F^−^, being Si the only element showing a negative correlation with As. If the Spearman correlation is used instead [34], other negative correlations can be observed, as for example with Mn, Cl^−^, SO_4_^2−^ and EC (at a 95% of confidence), and especially with Fe and Ca (at a 99% of confidence, *p* < 0.01).

### 3.2. Arsenic Detection with Field Kits

**Figure 3:**
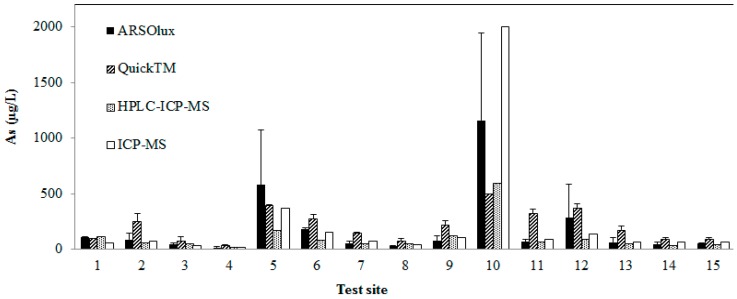
Arsenic concentrations detected with different methods (sample id map Figure 1: 1, 9-Rafaela; 2-Susana; 3,4-San José; 5-Sunchales; 6-Tacural; 7,8-dairy farm near Sunchales; 10-Bella Italia, dairy farm; 11-Bella Italia; 12-Bella Italia cemetery; 13,14-Presidente Roca; 15-School Presidente Roca). Data for ARSOlux and Quick^™^ represent means and standard deviation.

The arsenic results of the field test kits were in satisfying agreement with reference data determined by HPLC-ICP-MS (Figure 2 and Figure 3; Table A4), but the ARSOlux biosensor showed a much better correlation (r^2^ = 0.91, *n* = 15) than the Arsenic Quick^™^ field kit (r^2^ = 0.49, *n* = 15, Figure 2).

The arsenic speciation (AsV, AsIII) by HPLC-ICP-MS revealed average proportions of 98.5% arsenate and 1.5% arsenite of the total arsenic concentration, in agreement with previous results for groundwater of the Chaco-Pampean plain [5,6]. The average concentration determined by ICP-MS of 223 µg/L As (16–2000 µg/L) was higher than the result of HPLC-ICP-MS, averaging 108 µg/L (18–600 µg/L). Results of the ARSOlux biosensor and the Arsenic Quick^™^ test gave average arsenic concentrations of 190 µg/L (17–1161 µg/L) and 209 µg/L (35–500 µg/L), respectively. Results for average total arsenic concentration at the 15 different test sites around Rafaela evaluated by four different methods are shown in Figure 3. The analysis of ground water samples revealed that concentrations of several parameters of concern for drinking water quality were above the guideline values of WHO [16] and national guidelines (Table 2).

**Table 2:** Drinking water quality parameter * of 15 ground water samples taken near Rafaela, Santa Fe Province, Argentina (bold numbers indicate a concentration value above the guideline value *).

Spl. ID	As	Al	B	F	NO3^−^	Cl^−^	HCO3^−^	pH	EC
	µg/L	µg/L	mg/L	mg/L	mg/L	mg/L	mg/L		µS/cm
1	55	210	4.3	2.6	19.1	20.0	552.9	7.844	2730
2	73	123	5.4	1.3	131.1	94.2	967.6	7.991	3040
3	33	135	3.5	0.4	9.2	41.9	552.9	7.498	4040
4	16	114	4.3	0.6	638.5	304.5	1105.8	6.978	6900
5	370	103	2.7	1.1	15.4	127.5	992.7	8.3	1015
6	150	140	5.9	2.3	28.1	255.0	1130.9	7.872	2030
7	74	124	1.16	0.8	31.9	1208.3	754	7.69	906
8	41	115	4.5	n/a	n/a	n/a	1055.5	7.526	3910
9	103	98	4.7	0.7	31.5	296.8	716.3	7.984	2190
10	2000	114	8.6	0.5	39.6	196.0	791.7	8.436	3000
11	87	160	7.1	0.7	125.6	207.4	879.6	7.997	6950
12	140	133	4	2.6	19.1	20.0	552.9	7.872	1750
13	66	128	4.1	1.3	131.1	94.2	967.6	7.46	2880
14	63	490	4.9	0.4	9.2	41.9	552.9	7.49	2500
15	67	140	5.8	0.6	638.5	304.5	1105.8	7.667	3320

*: Guideline values: As: 10 µ/L (WHO, Argentina); Al: 0.2 mg/L (Argentina, Germany); B: 0.5 mg/L (Argentina), 1 mg/L (Germany); F: 1.5 mg/L (WHO); NO_3_^−^: 45 mg/L (Argentina), 50 mg/L (WHO); Cl^−^: 350 mg/L (Argentina), 250 mg/L (Germany); EC: 2750 µS/cm at 25 °C (Germany).

## 4. Discussion

### 4.1. Effects of the Chemical Composition of the Samples

According to Smedley and Kinniburgh [2], arsenic release can be triggered by high pH or by reducing conditions at near neutral pH. According to the obtained data and previous findings [5], the presence of arsenic in the studied region can be related with the alkaline conditions; in fact, a significant positive correlation was found between total arsenic and pH, as previously reported [5], although the measured pH values are lower than those that would be expected for a large arsenic release (pH > 8.5). Contrarily to previous findings, no correlations were found for bicarbonate and organic matter with total arsenic [2,7,17,35]. HCO_3_^−^ was considered to play a key role on arsenic presence, as it is formed by carbonate dissolution [7], it can compete with arsenate (As(V)) for adsorption sites [17,35] and can also participate in advanced silicate reactions [17]. Organic matter can generate anoxic conditions, especially during the dry season [36], which in turn can cause Fe(III) reduction and arsenic mobilization [2].

The rather high measured values of silicate (Si) have been related with the dissolution of volcanic glass [8,9], a process that was held responsible for the arsenic presence in the Chaco-Pampean plain [8,34] along with leaching of loess-type sediments [7]. Silicate is also known to have a positive correlation with total arsenic [2,34], as it competes with arsenic for adsorption sites in the aquifer [34]; the negative correlation here obtained with Si has been never reported before, although Gómez *et al.*, reported no correlation between total arsenic and Si [9]. The high values of Si can negatively affect the use of reverse osmosis for arsenic removal [37].

The positive correlations between total arsenic and V, F^−^ and B have been reported previously for ground waters in this region [2,6,7,8,17,34,35,36]. Also, the negative correlations of total arsenic with Fe and Ca have been observed before [2,34], as their presence should decrease As(V) concentration by precipitation and/or adsorption.

The extremely low values of arsenite (As(III)) concentration, near the detection limit of the technique, are in good agreement with the rather high concentrations of dissolved oxygen (DO > 3 mg/L), nitrate (NO_3_^−^ > 9 mg/L) and sulfate (SO_4_^2−^ > 550 mg L^−1^) [35], and the small concentrations of total iron (Fe tot < 0.20 mg/L); according to these values, As(III) formation should not be expected [2].

### 4.2. Comparison of the Technical Performance of Arsenic Field Test Kits

The results of the ARSOlux biosensor field kit were in better agreement with the results of the reference laboratory method (HPLC-ICP-MS, Figure 2) than the results of the Arsenic Quick^™^ kit. In our study the Arsenic Quick^™^ kit overestimated the arsenic concentration in most samples compared to the reference methods used (ICP-MS, HPLC-ICP-MS). Slightly higher total arsenic concentrations measured by ICP-MS compared to HPLC-ICP-MS in some of the samples can be explained by the fact that the ICP-MS samples are stabilized and digested with HNO_3_ leading to release of slight excess amounts of particulate arsenic to the solution. Arsenic associated with iron oxides cannot be re-dissolved completely by the mobile phase (HPLC-ICP-MS, eluent, diluted nitric acid), and therefore it can be retained on the stationary phase of the separation column.

The Quick^™^ kit delivers only semi-quantitative data compared to distinct readings of the luminometer device used in the biosensor test kit. Several hazardous chemicals such as mercury bromide (HgBr_2_) are used in the chemical test kit; the wastes including the sample/reagent mixture (100 mL for each sample) must be collected after the test and have to be disposed properly. During the testing process, highly toxic arsine gas (AsH_3_) is generated, which potentially threatens the health of the test kit user. An advantage of the Quick^™^ kit is its comparably simple and fast handling and small size. Yet, it yields inaccurate results thus misleading the users and potentially threatening their health. The biosensor is environmentally friendly, does not need toxic reagents and yields accurate quantitative results. Very little wastes are generated by the biosensor since only 1 mL of sample is required for the testing process. The biosensor is particularly suitable for the screening of a high number of wells, whereas it requires more preparation effort and time compared to the chemical test kit when only one or two samples have to be tested. If a high number of samples of up to 100 have to be screened regularly, the biosensor is clearly superior in terms of time and material efficiency.

### 4.3. Considerations for Deployment of the Arsenic Biosensor ARSOlux

The deployment of ARSOlux is suitable for monitoring and screening programs in Argentina and in other Latin American and Asian countries. It would allow the authorities and non-government agencies of severely arsenic affected regions to monitor arsenic in drinking water wells and treatment plants at much lower costs and in a more practical manner than with conventional laboratory and field test methods. ARSOlux thus holds promise as element of comprehensive solutions for the mitigation of the arsenic problem in countries where means and resources to supply arsenic free water to all citizens are lacking. It is crucial for the successful implementation of ARSOlux to obtain permits for export and import as well as a license for distribution from the local governmental entities. The import of genetically modified organisms (GMO) to user countries is regulated by international and national laws and guidelines based on the Cartagena Protocol on Biosafety [38]. A risk assessment prepared by the Central Commission on Biologic Safety (ZKBS) of the ARSOlux biosensor for usage in the frame of a research campaign was published at websites of the Biosafety Clearing-House and the ZKBS, Germany [39]. The biosensor contains a non-pathogenic *Escherichia coli* DH5α strain. Guidelines and procedures for import permits for research or commercial use vary widely globally. The processing time for applications can take several months or even years.

At present, the use of the bioreporter bacteria in the ARSOlux kit, which belong to the *Escherichia coli* K12 group, is restricted to laboratories equipped according to WHO biosafety level I [40]. In Saxony, Germany the biosensor has been used in a mobile laboratory installed in a van, with permission of the Saxon State Ministry of the Environment and Agriculture (SMUL). The mobile lab was registered as a genetic engineering facility equipped with manuals, safety sheets and basic protection as well as deactivation and disposal materials such as disinfection flasks and autoclaving containers. A similar mobile lab can be recommended for regions in India and Bangladesh, where large numbers of wells or other water sources have to be tested in a comparably small area. Contrary to that, ground water wells and other water sources in regions such as the Chaco-Pampean Plains are relatively distant from each other. The sampling of vast amounts of wells can take several days or weeks. It is most efficient to collect a certain number of samples and to store them in the laboratory. After a certain number of samples are collected then the analysis with the biosensor test kit is executed within about two and a half hours in a closed laboratory (e.g., research institute, government agency, lab van) at a central location. It is important to filter the samples and store them in a refrigerator to preserve the original composition and avoid co-precipitation processes. Further investigations are going to show if the freezing of samples can be recommended for long time storage.

Generally, biosensors can be used as an alternative to expensive spectrometric devices or other high-end laboratory equipment. In rural areas of Argentina it is not always required to test for the full range of water quality parameters at the highest possible accuracy. Hence, the ARSOlux biosensor can serve as a cost effective and simple tool yielding results for one specific parameter. The cost for an ICP-MS device for example amounts to about 120,000 to 300,000 Euros. The ARSOlux biosensor test kit included in a multiple parameter testkit with photo- as well as luminometer device might only cost approximately 4000 Euro, while a single biosensor test for arsenic will cost approximately 2 Euro. While stationary spectrometric devices need a supply of chemicals, gases and power, biosensor tests can be run by a battery driven device, which is easily transportable. This is important since in many targeted user countries, power outages are a serious issue. By using biosensor test kits such as ARSOlux, it could be possible to guarantee continuous monitoring of water quality also in regions of Argentina and Latin America where limited resources and infrastructure complicate the distribution of safe drinking water.

## 5. Conclusions

The deployment of the ARSOlux biosensor under real conditions is a step forward to demonstrate that advanced bio-reporter systems for detection of environmental arsenic contamination could be of great benefit to mitigate health issues related to arsenic in drinking water. It is the first time that a biosensor has been used in the described context in Latin-America. A cost-effective and simple biosensor for detection of bioavailable arsenic could replace expensive, complicated and inaccurate analytical methods to guarantee better living standard also in less developed regions. Biosensors containing recombinant bio-reporter bacteria could not yet be transferred into real markets because of legal challenges. Nevertheless it is of great importance to step forward to make them available to water industries and society, improve environment, well-being and health of the rural population. The results of the present study show that the biosensor test kit is robust enough and accurately detects the total bioavailable arsenic concentration also under specific conditions of rural Argentina. Previous findings detecting arsenate as the predominant species in ground water in the Chaco-Pampean plain were confirmed.

## Appendix

**Table A1:** GPS location of 15 groundwater sampling sites near Rafaela, Santa Fe province, Argentina (Garmin eTrex 10 GPS device, Garmin, Southampton, U.K.).

Spl. ID	Latitude S	Longitude W
1	31.273594	61.489766
2	31.357068	61.513025
3	31.338499	61.622651
4	31.338839	61.622951
5	30.943634	61.556958
6	30.845649	61.590777
7	31.029629	61.454898
8	31.029501	61.454980
9	31.271950	61.500500
10	31.314193	61.363348
11	31.296873	61.390786
12	31.269692	61.431587
13	31.215263	61.616112
14	31.215168	61.616238
15	31.232784	61.609028

**Table A2:** Water quality data of 15 groundwater samples taken near Rafaela, Santa Fe province, Argentina (on-site measurements with multi parameter probe (WTW, Multi 3430 SET G, Weilheim, Germany).

Spl. ID	pH	EC µS/cm	DO mg/L	TDS mg/L	Sal	T °C
1	7.844	2730	6.89	1908	1.4	20.4
2	7.991	3040	6.79	2120	1.6	18.8
3	7.498	4040	7.35	2930	2.1	18.7
4	6.978	6900	7.03	4830	3.8	20.3
5	8.3	1015	4.73	711	0.4	20.7
6	7.872	2030	6.25	1421	1	19.5
7	7.69	906	4	634	0.4	20.8
8	7.526	3910	6.9	2730	2	19
9	7.984	2190	8.73	1534	1.1	19.1
10	8.436	3000	3.72	2100	1.5	20.7
11	7.997	6950	3.28	4860	3.8	20
12	7.872	1750	8.3	1224	0.8	20
13	7.46	2880	5.84	1978	1.4	18
14	7.49	2500	5.66	1754	1.3	20
15	7.667	3320	8.4	2330	1.7	13.2

**Table A3:** Arsenic concentrations of 15 groundwater samples taken near Rafaela, Santa Fe province, Argentina (ARSOlux biosensor measured at UTN lab Rafaela, Arsenic Quick^™^ measured on site, HPLC-ICP-MS, ICP-MS measured at UFZ lab, Leipzig, Germany).

Spl. ID	ARSOlux	Arsenic Quick^™^	HPLC-ICP-MS		
	As tot	As tot	As tot	As III	As V
	µg/L				
1	110.5	100	116.9	1.4	115.4
2	88.4	250	60.8	0.9	59.9
3	43.5	75	50.5	1.2	49.3
4	17.3	35	18.4	0.6	17.9
5	579.3	400	174.1	0.7	173.4
6	182.0	275	83.5	0.7	82.8
7	55.2	150	51.9	0.5	51.5
8	30.0	75	54.4	1.0	53.4
9	77.0	225	124.2	0.5	123.7
10	1160.8	500	599.7	0.6	599.1
11	68.9	325	66.0	1.1	65.0
12	284.0	375	89.5	1.2	88.2
13	61.9	175	53.4	1.0	52.4
14	44.4	90	38.0	1.3	36.7
15	51.2	90	43.9	1.0	42.9

**Table A4:** Water quality data of 15 groundwater samples taken near Rafaela, Santa Fe province, Argentina (ICP-MS, UFZ, Leipzig, Germany).

Spl. ID	As	Al	Cr	Fe	Mn	V	B	Ca	Si
	µg/L						mg/L		mM
1	55	210	10.4	109	5.7	160	4.3	17	1.21
2	73	123	4.3	50	1.5	150	5.4	45	1.39
3	33	135	3	34	2.6	102	3.5	123	1.42
4	16	114	4.9	66	1.6	76	4.3	290	1.50
5	370	103	1.3	4.7	0.25	550	2.7	4.2	1.17
6	150	140	1.1	9.2	0.99	290	5.9	22	1.25
7	74	124	1	21	0.35	150	1.16	39	1.25
8	41	115	3	29	6.5	113	4.5	98	1.39
9	103	98	8	12.3	0.21	180	4.7	16	1.25
10	2000	114	5.4	3.6	0.14	1090	8.6	4.3	1.10
11	87	160	4.3	44	3.9	190	7.1	48	1.17
12	140	133	5.1	18	0.61	230	4	14	1.35
13	66	128	5.1	15	0.126	133	4.1	53	1.39
14	63	490	5.3	160	23	160	4.9	47	1.46
15	67	140	2.7	132	5.6	160	5.8	62	1.32

**Table A5:** Water quality data of 15 groundwater samples taken near Rafaela, Santa Fe province, Argentina (UTN, Rafaela and Buenos Aires, Argentina).

Spl. ID	F	NO_3_^−^	Cl^−^	HCO_3_^−^	SO_4_^2^^−^	Total Carbon	Inorganic Carbon	Total Organic Carbon
	mg/L					mg∙C/L		
1	2.6	19.1	20.0	552.9	32.6	200.5	158.4	42.1
2	1.3	131.1	94.2	967.6	115.1	220.3	195.0	25.4
3	0.4	9.2	41.9	552.9	103.2	184.9	126.5	58.4
4	0.6	638.5	304.5	1105.8	340.9	170.6	111.4	59.2
5	1.1	15.4	127.5	992.7	220.7	150.1	97.2	53.0
6	2.3	28.1	255.0	1130.9	439.4	200.6	173.7	26.9
7	0.8	31.9	1208.3	754	1091.3	137.4	96.4	41.1
8	n/a	n/a	n/a	1055.5	109.1	221.8	197.6	24.2
9	0.7	31.5	296.8	716.3	453.8	198.3	160.3	38.0
10	0.5	39.6	196.0	791.7	396.2	219.8	199.1	20.8
11	0.7	125.6	207.4	879.6	596.2	180.5	133.8	46.7
12	2.6	19.1	20.0	552.9	32.6	218.9	185.3	33.6
13	1.3	131.1	94.2	967.6	115.1	179.1	124.7	54.5
14	0.4	9.2	41.9	552.9	103.2	189.7	141.7	48.0
15	0.6	638.5	304.5	1105.8	340.9	198.3	150.0	48.3
